# Improved privacy preserving method for periodical SRS publishing

**DOI:** 10.1371/journal.pone.0250457

**Published:** 2021-04-22

**Authors:** Wei Huang, Tong Yi, Haibin Zhu, Wenqian Shang, Weiguo Lin

**Affiliations:** 1 Division of Scientific Research, Communication University of China, Beijing, China; 2 School of computer information and Engineering, Guangxi Normal University, Guilin, Guangxi, China; 3 Department of Computer Science and Mathematics, Nipissing University, North Bay, Ontario, Canada; 4 School of Computer Science, Communication University of China, Beijing, China; University of Management and Technology, PAKISTAN

## Abstract

Spontaneous reporting systems (SRSs) are used to collect adverse drug events (ADEs) for their evaluation and analysis. Periodical SRS data publication gives rise to a problem where sensitive, private data can be discovered through various attacks. The existing SRS data publishing methods are vulnerable to Medicine Discontinuation Attack(*MD*-attack) and Substantial symptoms-attack(*SS*-attack). To remedy this problem, an improved periodical SRS data publishing—PPMS(*k*, *θ*, *ɑ*)-bounding is proposed. This new method can recognize *MD*-attack by ensuring that each equivalence group contains at least *k* new medicine discontinuation records. The *SS*-attack can be thwarted using a heuristic algorithm. Theoretical analysis indicates that PPMS(*k*, *θ*, *ɑ*)-bounding can thwart the above-mentioned attacks. The experimental results also demonstrate that PPMS(*k*, *θ*, *ɑ*)-bounding can provide much better protection for privacy than the existing method and the new method dose not increase the information loss. PPMS(*k*, *θ*, *ɑ*)-bounding can improve the privacy, guaranteeing the information usability of the released tables.

## 1. Introduction

Many developed countries have established spontaneous reporting systems (SRSs) for the collection of adverse drug events (ADEs). These datasets allow researchers to analyze possible correlations between drugs and adverse reactions. Typical spontaneous reporting systems include FAERS of the US Food and Drug Administration [1] and the UK Yellow Card scheme [2].

However, these datasets usually involve information which relates to an individual’s privacy. Sensitive attributes (SAs), e.g., adverse drug reaction and disease type are also included. Publishers usually remove attributes which can identify individuals uniquely before releasing their reports, however, Sweeney [3] has pointed out that an adversary can use quasi-identification attributes (QIAs) to link the released table to other publicly available datasets in an effort to uniquely identify an individual. A QIA can be Age, Gender, etc. A single quasi-identification attribute cannot uniquely identify an individual. To protect SAs, privacy preserving data publishing (PPDP) usually anonymizes original tables before releasing. In recent years, PPDP has been widely studied and seeks to maintain the tradeoff between privacy/security and information usability in released tables. *K*-anonymity [3] and its variants [4–8] are only suitable for static tables. When faced with Dynamic data tables, some incremental data publishing methods [9–14] are presented, such as *BCF*-anonymity [9], *m*-invariance [10], etc. Most techniques cannot preserve identity in released tables and are not well suited to SRS data publishing. Differential privacy [15–18] can make presence or absence of a record in the dataset have little effect on the outcome. However, the utility of released tables will be adversely affected by the added noise [19].

SRSs release updated datasets periodically, for example, the US Food and Drug Administration releases the adverse drug event datasets quarterly. Lin et al. [20–21] showed that SRS datasets usually include some characteristics, e.g., multiple individual records, multivalued sensitive attributes, etc. More importantly, the related ADE records of an individual may be contained in tables released in each period. These records share case identification (CaseID) to trace follow-ups to an event [22]. Thus, conventional data publishing methods cannot handle SRS datasets. To resolve this, Wang et al. [22] defined three types of attacks in SRS dataset publishing and presented a periodical SRS data publishing method- PPMS(*k*, θ*)-bounding. These attack types are defined as follows.

### Definition 1 (Backward-attack)

Assume target individual *P* whose record *t* is in sanitized released table *T*_*i*_, use *t*.*QIA* and *C* to represent the QIAs values of *t* and *P*’s candidate CaseID set in *T*_*i*_, respectively. *U* contains all this record *r*: *r* is from the previous released tables {*T*_1_, *T*_2_, …, *T*_*i*-1_}, and *r*’ s CaseID is in *C*. The Backward-attack (*B*-attack) may happen if there is a record *r* (*r*∈*U*) whose QIAs values *r*.*QIA* does not cover *t*.*QIA*. We Denote the set of these excludable records as *B*. The QIAs values of *r* (*r*.*QIA*) cover the ones of *t* (*t*.*QIA*), if the records *r* and *t* satisfy: for each quasi-identification attribute *QI* in *QIA*, *r*.*QI* is equal to or more generalized than *t*.*QI*.

### Definition 2 (Forward-attack)

Assume target individual *P* whose record *t* is in sanitized released table *T*_*i*_. We use *t*.*QIA* and *C* to represent the QIAs values of *t* and *P*’s candidate CaseID set in *T*_*i*_, respectively. *U* contains all this record *r*: *r* is from the subsequent released tables {*T*_*i*+1_, *T*_*i*+2_, …}, and *r*’s CaseID is in *C*. The Forward-attack(*F*-attack) may happen if there is a record *r* (*r*∈*U*) whose QIAs value *r*.*QIA* does not cover *t*.*QIA*. Denote the set of these excludable records as *F*.

### Definition 3 (Latest-attack)

Assume target individual *P* whose record *t* is in sanitized released table *T*_*i*_, and none of the previous released tables {*T*_1_, *T*_2_, …, *T*_*i*-1_} contain the CaseID of *P*. We use *C* to represent the *P*’s candidate CaseID set in *T*_*i*_. The Latest-attack(*L*-attack) may happen if there is any CaseID *cid*(*cid*∈*C*) which appears in some previous released tables. Denote the set of these excludable records as *L*.

Based on the above three attacks, the definition of an anonymity model can be introduced [22]:

### Definition 4 (PPMS(*k*, θ*)-bounding)

Assume {*s*_1_, *s*_2_, …, *s*_*m*_} are all the possible sensitive attribute values in datasets, accordingly, θ* = {*θ*_1_, *θ*_2_, …, *θ*_m_} are the probability thresholds set by the data holder. Released tables *T*_1_, *T*_2_, …, *T*_*j*_ satisfy PPMS(*k*, θ*)-bounding if each *T*_*i*_ (1≤*i*≤*j*)satisfies:

1. For each individual *P*, assume the candidate CaseID set of *P* in *T*_*i*_ is *C*, then there is |*C*-(*B*∪*F*∪*L*)|≥*k*;
2. For every individual *P*, an adversary can conclude that *P* has any sensitive attribute value *s*_*j*_ with a probability at most *θ*_*j*_.

PPMS(*k*, θ*)-bounding uses *NC*-bounding and *OID*-bounding to defend against the above three attacks. *NC*-bounding makes each group contain at least *k* new CaseIDs, which can defend against backward-attack and latest-attack. Let *t* be a record in released *T*_*i*_, *t*_1_, *t*_2_, …, *t*_*j*_ are records have the same CaseID with *t* in previous tables *T*_x_, *T*_y_, …, *T*_*z*_(*x*<*y*<…, <*z*). To thwart forward-attack, *OID*-bounding requires that QIAs values of *t* should cover all records that share the same CaseID with *t* in previous tables. Wang et al. [22] also found that the forward-attack can be avoided when QIAs values of *t* only cover the records that share the same CaseID with *t* in *T*_*x*_, they call this method PPMS_EAR. Their experiments show that PPMS_EAR can thwart the above three attacks while maintaining the usability of tables.

Table 1(A)–1(C) are three original tables, Adverse Drug Reaction (ADR) is a sensitive attribute, and Sex and Age are quasi-identification attributes. For simplicity, letters are used to denote a type of ADR. Table 2(A)–2(C) are corresponding sanitized released tables which satisfy PPMS(3, 1/3)-bounding. Obviously, the released tables can thwart backward-attack, forward-attack and latest-attack. However, an adversary can still disclose the privacy of individuals. Let us consider some examples.

**Table 1:** Three consecutive quarters of original SRS dataset.

(a) Quarter 1			
CaseID	Sex	Age	ADR
1	M	50	c,b
7	M	48	a
3	M	46	d
5	M	46	e,g
2	F	21	c,a
4	F	23	b,d
6	F	25	y
(b) Quarter 2			
CaseID	Sex	Age	ADR
1	M	50	c,b
11	M	50	a
12	M	53	y
14	M	48	h
16	M	48	q,d,e,p,x
15	F	40	x
18	F	39	q
17	M	46	y,b,c,f,g,i
19	F	43	o
3	M	46	d
20	F	40	x,i
21	F	46	j,z,v,u,k,n
22	M	46	q,l
13	F	39	h,o
(c) Quarter 3			
CaseID	Sex	Age	ADR
13	F	40	h,k
26	F	45	x,u
28	F	45	d,i
23	F	40	z
15	F	39	x
27	M	38	a,c
24	M	38	d
25	M	38	e,q

**Table 2:** PPMS(3,1/3)-bounding publishes tables for Table 1.

(a) Quarter 1				
CaseID	Sex	Age	ADR	Group
1	M	[46–50]	c,b	1
7	M	[46–50]	a	1
3	M	[46–50]	d	1
5	M	[46–50]	e,g	1
2	F	[21–25]	c,a	2
4	F	[21–25]	b,d	2
6	F	[21–25]	y	2
(b) Quarter 2				
CaseID	Sex	Age	ADR	Group
1	M	[48–53]	c,b	1
11	M	[48–53]	a	1
12	M	[48–53]	y	1
14	M	[48–53]	h	1
13	F	[39–40]	h,o	2
15	F	[39–40]	x	2
18	F	[39–40]	q	2
16	*	[46–48]	q,d,e,p,x	3
17	*	[46–48]	y,b,c,f,g,i	3
21	*	[46–48]	j,z,v,u,k,n	3
3	*	[40–46]	d	4
20	*	[40–46]	x,i	4
19	*	[40–46]	o	4
22	*	[40–46]	q,l	4
(c) Quarter 3				
CaseID	Sex	Age	ADR	Group
13	F	[39–45]	h,k	1
26	F	[39–45]	x,u	1
28	F	[39–45]	d,i	1
23	F	[39–45]	z	1
15	*	[38–40]	x	2
27	*	[38–40]	a,c	2
24	*	[38–40]	d	2
25	*	[38–40]	e,q	2

### Example 1

Bob knows that Alice(F, 39) is in Table 2(B), and he can relate Alice to records{*t*_13_, *t*_15_, *t*_18_}. He also knows that Alice will stop medicine in quarter 3, because her illness is shown as cured in quarter 2. Therefore, Bob can exclude *t*_13_, *t*_15_, and conclude Alice’s record is *t*_18_ with the probability of 100%. The privacy of Alice is disclosed.

### Example 2

Bob knows that Clare(M, 47) is in Table 2(B), and can be related to records{*t*_16_, *t*_17_, *t*_21_}. Bob cannot relate Clare to a unique record, but {*t*_16_, *t*_17_, *t*_21_} all contain many more adverse drug reactions than other records. Thus, Bob can conclude that Clare gets more adverse drug reactions than other people. The privacy of Clare is disclosed.

PPMS(*k*, θ*)-bounding has not considered medicine discontinuation and records with massive symptoms, hence privacy may be disclosed by an adversary. As the extended versions of PPMS(*k*, θ*)-bounding, the other existing SRS data publishing methods [23–25] have not considered the attacks described by example 1 and example 2, either. It is necessary to find a way to defend against the above attacks. However, increasing the security usually makes the information usability decline. It is challenging to balance privacy security against the information utility. To alleviate these problems, this paper proposes a new SRS data publishing method which can improve the privacy and guarantee the information usability. The main contributions of this paper are summarized as follows:

1. Identifying two new attacks which are aimed at ADEs data publishing;
2. Based on the PPMS(*k*, θ*)-bounding, proposing a new data publishing method- PPMS(*k*, *θ*, *ɑ)*-bounding. The new method can enhance the security of privacy and preserve the quality of released tables. A corresponding algorithm is presented.
3. Using a real FAERS database from the US Food and Drug Administration to verify PPMS(*k*, *θ*, *ɑ*)-bounding.

## 2. Related work

ADE reporting is a special style of incremental data publishing released periodically. Subsequent tables may add new records, and delete/update records offered previously. For the purpose of tracing individuals, the same CaseID can appear in different released tables. Wang et al. [22] divided traditional incremental data publishing into two types: continuous data publishing and dynamic data publishing.

Continuous data publishing [9, 11]: periodic publishing that carries over records from previously released tables. The data holder needs to release all the data collected so far, if he wants to publish the data which is collected recently. Suppose that the data holder has collected data *D*_*i*_ in timestamped *t*_*i*_. In general, the data holder has to release *R*_*i*_ which is the anonymized version of *D*_1_∪*D*_2_∪…*D*_*i*_ in timestamped *t*_*i*_.

Some matching records can be excluded by the adversary, because he/she can infer that the records are not related to the target’s QIA values or timestamp [9]. Thus, Fung et al. [9] pointed out that the excluded records can help the adversary access to a smaller set of candidates. Thus, they presented a privacy model (called *BCF*-anonymity) to evaluate anonymity after excluding some matching records. Besides, an efficient algorithm was presented to achieve a suboptimal *BCF*-anonymization.

Pei et al. [11] pointed out that in continuous data publishing scenario, *k*-anonymity [3] may be compromised due to the possible inferences using multiple releases. They presented a privacy preserving approach, called Monotonic Incremental Anonymization, to guarantee the *k*-anonymity on each release. Meanwhile, the approach can reduce information loss by using more and more accumulated data.

Some continuous data publishing methods can preserve the identities of individuals among different tables [11], but this type of methods cannot support the operations of deletion and updating. Therefore, dynamic data publishing methods were presented later.

Dynamic data publishing [10, 12–14]: periodic publishing where records can be added, deleted or updated from previously released tables. This method cannot preserve the identities of individuals among different tables. Suppose that the data holder had collected the initial set of tuples *D*_1_ in time *t*_1_, and published *R*_1_ as the anonymized version of *D*_1_. During the period [*t*_1_,*t*_2_], when there were new records coming, the data holder inserted them into *D*_1_. At the same time, some records from *D*_1_ might be deleted or updated by the data holder. Finally, the *D*_2_ could be obtained in time *t*_2_. Thus, the data holder published *R*_2_ as the anonymized version of *D*_2_. In general, the data holder publishes *R*_*i*_ as the anonymized version of *D*_*i*_ in time *t*_*i*_.

Xiao et al. [10] found out that when incremental data publishing supported deletions, the adversary could disclose the privacy of victims by comparing the series of released *k*-anonymous [3] and *l*-diverse [8] data. They presented a privacy model, called *m*-invariance, to guarantee certain “invariance” in all the QIA groups that a tuple is incorporated into at different publication timestamps.

Li and Zhou [12] defined the updates on attribute values as internal updates. They pointed out that the internal updates related to sensitive values were not arbitrary, the requirement of *m*-invariance was unreachable in this scenario. A counterfeit generalization approach called *m*-Distinct was presented to guarantee the security of dynamic publication with internal updates, insertions and deletions.

Following the work of [12], Anjum and Raschia [13] further assumed that new values might not have any association with the old ones, and the adversary knew the “event list”. An attack model based on their assumption, called *τ*- attacks, was defined. To prevent the new attack, Anjum and Raschia also presented a publication approach called *τ*-safety, which is based on *m*-invariance and individual-oriented protection.

Bewong et al. [14] illustrated that the transactional data had some special features, such as having many common private terms. Thus, they pointed out that the existing incremental publishing methods were inapplicable. A transactional data publication mechanism called Sanony was also presented, to prevent composition attacks by utilizing counterfeits.

Fully data evolution is supported in dynamic data publishing. However, identity preservation cannot be supported in this type of multiple releases, which results in its inapplicability to ADEs data publishing.

Differential privacy [15–18] has garnered a lot of attention in recent years, it can minimize the chances of identifying records. However, the noise added by differential-based methods is unbounded and random, which will adversely affect the utility of released tables [19].

2016, Lin et al. [21] began to study ADEs data publishing, and presented the MS(*k*, θ*)-bounding method based on the characteristics of ADE data. Because this method had not considered the correlation among different released tables, an adversary can exploit this situation when seeking to disclose the privacy of individuals. To resolve this, 2017 Wang et al. [22] proposed PPMS(*k*, θ*)-bounding for periodical ADEs data publishing. This method can defend against the three attack methods(*B*-attack, *F*-attack and *L*-attack) which are based on correlations among different released tables. After that, several ADEs data publishing methods based on PPMS(*k*, θ*)-bounding were presented [23–25]. Hsiao et al. [23] presented a privacy model, called Closed *l*-diversity, to process the missing value by guaranteeing that each partial QID-group includes at least *l* different sensitive values. They also proposed an algorithm, called Closed *l*-diversification, to achieve Closed *l*-diversity. Cui et al. [25] presented a SRSs data publication approach, called EQZS, to improve the efficiency of PPMS(*k*, θ*)-bounding. The new values and old values covered each other in this method, which resulted in the limitation of the released data usability.

The existing SRSs data publishing methods have not considered the situation of medicine discontinuation and massive symptoms. The adversary can use related background knowledge to disclose privacy. This paper presents a new ADEs data publishing method to address the matter.

## 3. PPMS(*k*, *θ*, *ɑ*)-bounding model

In PPMS(*k*, θ*)-bounding [22], the adversary learns target individual *P*’s QIAs values, and knows *P* in a released table. An initial adverse drug reaction can also be revealed. We assume the adversary may learn extra information: *P* stops medication in the next quarter. Patients will stop the medication when the illness is cured or other therapies(e.g., surgery, food therapy) are chosen. Thus, the assumption is realistic. The adversary can use the information of medication discontinuation to disclose the privacy, like in example 1.

### Definition 5 (Medication Discontinuation-attack)

Assume target individual *P* whose record *t* is in sanitized released table *T*_*i*_, and *T*_*i*+1_ does not contain the CaseID of *P*. Use *C* to represent the *P*’s candidate CaseID set in *T*_*i*_. The Medication discontinuation**-**attack(*MD*-attack) may happen if there is any CaseID *cid*(*cid*∈*C*), which appears in *T*_*i*+1_. Denote the set of these excludable records as *MD*. Example 1 is an instance of *MD***-**attack.

### Definition 6 (Substantial Symptoms-attack)

The adversary can conclude that the target individual experiences more symptoms/adverse drug reactions than other people. We have used example 2 to illustrate this style of attack (*SS*-attack). We refer to the record with substantial symptoms/adverse drug reactions as an **ss-record**. Publishers can decide the specific method for defining an ss-record.

Based on PPMS(*k*, θ*)-bounding, PPMS(*k*, *θ*, *ɑ*)-bounding needs to thwart these two new attacks:

### Definition 7 (PPMS(*k*, *θ*, *ɑ*)-bounding)

Assume {*s*_1_, *s*_2_, …, *s*_*m*_} are values of all the possible sensitive attributes in the datasets, accordingly, *θ* = {*θ*_1_, *θ*_2_, …, *θ*_m_} are the probability thresholds set by the data holder. Released tables *T*_1_, *T*_2_, …, *T*_*j*_ satisfy PPMS(*k*, *θ*, *ɑ*)-bounding if each *T*_*i*_ (1≤*i*≤*j*)satisfies:

1. *k*-bounding: For each individual *P*, assume that the candidate CaseID set of *P* in *T*_*i*_ is *C*, then there is |*C*-(*B*∪*F*∪*L*∪*MD*)|≥*k*;
2. *θ*-bounding: For every individual *P*, an adversary can conclude that *P* has any sensitive attribute value *s*_*j*_ with a probability at most *θ*_*j*_.
3. *a*-bounding: For every individual *P*, an adversary can conclude that *P* has many more symptoms/adverse drug reactions than others with the probability at most *ɑ*.

The privacy requirement of Definition 7(1) is used to avoid record disclosure. *MD*-attack is considered under this requirement which is the extended version of PPMS(*k*, θ*)-bounding. The adversary cannot distinguish the target individual from at least *k* records, even though he/she has excluded some candidates through *MD*-attack、*F*-attack、*B*-attack and *L*-attack. The privacy requirement of Definition 7(2) states that the probability of attribute disclosure will not exceed a threshold, even though the adversary can exclude some candidate records from various attacks. Thus, this privacy requirement is to guarantee the security of sensitive attributes values. Besides, the *SS*-attack can be thwarted by meeting the privacy requirement of Definition 7(3) that limits the frequency of ss-record in groups.

To satisfy the PPMS(3, 1/3, 1/4)-bounding, Table 3(A)–3(C) can be released for Table 1(A)–1(C). Each sanitized group incorporates at least three such individual *P* which have these properties: (a) *P* is the first time appearing in multiple releases; (b) *P* will not appear in the next release. At the same time, the new sensitive values cover the corresponding old ones according to the *OID*-bounding. Each group still contains at least three records and the frequency of each sensitive value does not exceed the threshold 1/3, even though the sets *B*,*F*,*L* and *MD* have been excluded by the adversary. Thus, the attacks in [22] can be prevented. Specially, the exclusion of set *MD* from releases has no effect on reaching privacy acquirements, so *MD*-attack in example 1 can be resisted. Besides, the frequency of ss-record in groups does not exceed 1/4, the *SS*-attack (example 2) can be also thwarted.

**Table 3:** PPMS(3,1/3,1/4)-bounding publishes tables for Table 1.

(a) Quarter 1				
CaseID	Sex	Age	ADR	Group
1	M	[46–50]	c,b	1
7	M	[46–50]	a	1
3	M	[46–50]	d	1
5	M	[46–50]	e,g	1
2	F	[21–25]	c,a	2
4	F	[21–25]	b,d	2
6	F	[21–25]	y	2
(b) Quarter 2				
CaseID	Sex	Age	ADR	Group
1	M	[48–53]	c,b	1
11	M	[48–53]	a	1
12	M	[48–53]	y	1
14	M	[48–53]	h	1
16	M	[48–53]	q,d,e,p,x	1
15	*	[40–46]	x	2
18	*	[40–46]	q	2
17	*	[40–46]	y,b,c,f,g,i	2
19	*	[40–46]	o	2
3	*	[39–46]	d	3
20	*	[39–46]	x,i	3
21	*	[39–46]	j,z,v,u,k,n	3
22	*	[39–46]	q,l	3
13	*	[39–46]	h,o	3
(c) Quarter 3				
CaseID	Sex	Age	ADR	Group
13	F	[39–46]	h,k	1
26	F	[39–46]	x,u	1
28	F	[39–46]	d,i	1
23	F	[39–46]	z	1
15	*	[38–46]	x	2
27	*	[38–46]	a,c	2
24	*	[38–46]	d	2
25	*	[38–46]	e,q	2

## 4. Algorithm and analysis

In this section, we propose an heuristic algorithm to achieve PPMS(*k*, *θ*, *ɑ*)-bounding. The related definitions and symbols are in the section 4.1. We give specific steps and illustration of the algorithm in section 4.2, meanwhile, the analysis and lemmas on which the algorithm depends are also included.

### 4.1 Definitions and symbols

Before stating the algorithm, we introduce new symbols as follows:

Substantial symptoms-record(ss-record): as mentioned earlier, for a record *t*(*t*∈*T*), if *t* has many more symptoms/adverse drug reactions than others in *T*, then *t* is an ss-record in table *T*.

New-record (n-record): for a record *t*(*t*∈*T*), *t*’s *caseid* is its initial appearance in a released table. That is, *t* is a n-record in table *T*.

Old-record(o-record): for a record *t*(*t*∈*T*), *t*’s *caseid* is not the initial appearance in a released table. That is, *t* is an o-record in table *T*.

Medication discontinuation-record(md-record): for a record *t*(*t*∈*T*_*i*_), *t*’s *caseid* will not appear in next table *T*_*i*+1_, *t* is a md-record in *T*_*i*_.

n*x*-record(*x*∈{ss, n, o, md}): if *t* is not *x* kind of record, *t* is n*x*-record. For instance, if *t* is not a md-record in table *T*, then *t* can be denoted as nmd-record in *T*.

*x*&*y*-record(*x*, *y*∈{ss, n, o, md}): if *t* is *x* kind of record and *y* kind of record, then *t* is an *x*&*y* -record. For instance, if *t* is a n-record and md-record in table *T*, then *t* can be denoted as n&md-record in *T*.

According to the background knowledge, the adversary can derive four views for an anonymous group *G*. Assume the adversary knows the target individual *P*’s QIAs values, and learns that the target is in a released table.

View 1: the adversary knows that individual *P* is in a specific group *G*, *P* appears for the first time in released tables and *P* stops medication in next quarter. We denote view 1 of group *G* as *GV*_1_. *GV*_1_ contains all the n&md-records in *G*.
View 2: the adversary knows that individual *P* is in a specific group *G*, *P* appears for the first time in released tables. We denote view 2 of group *G* as *GV*_2_. *GV*_2_ contains all the n-records in *G*.
View 3: the adversary knows that individual *P* is in a specific group *G*, *P* stops medication in next quarter. We denote view 3 of group *G* as *GV*_3_. *GV*_3_ contains all the md-records in *G*.
View 4: the adversary knows *P* is in a specific group *G*. We denote view 4 of group *G* as *GV*_4_. Obviously, *GV*_4_ = *G*.
*GV*_*x*_(*y*) (*x*∈{1, 2, 3, 4}, *y*∈{ss, n, o, md}): represents the set of all the *y* type of records in *GV*_*x*_.
F_*ss*_(*GV*_*x*_) (*x*∈{1, 2, 3, 4}): the frequency of ss-record in *GV*_*x*_.
If the adversary can disclose privacy in any one of these views, the anonymous group *G* is unsafe. Thus, we have to provide privacy protection in all these four views.

### 4.2 Algorithm of PPMS(*k*, *θ*, *ɑ*)-bounding

We present an algorithm called **HA** to achieve PPMS(*k*, *θ*, *ɑ*)-bounding. The overview of this algorithm is as shown in Algorithm 1.

**Algorithm 1: HA**

**Input**: the original dataset *D*_*i*_, the previous anonymous released tables *T*_*pre*_ = {*T*_1_, *T*_2_, …, *T*_*i*-1_}, *k*, *θ*, *α*

**Output**: anonymous released table *T*_*i*_ of original table *D*_*i*_

1:Combine records with the same caseid into a super record;

2:**for each** o-record *t* in *D*_*i*_ **do**

3:{

4: Find a released table *T*_*j*_ which is the first table contains caseid of *t* in *T*_*pre*_;

5: Record *t*_*pre*_ (*t*_*pre*_∈*T*_*j*_) has the same caseid with *t*, generalize the QIA values of *t* to cover that of *t*_*pre*_;

6:}

7:Grouping(*T*_*i*_, *D*_*i*_, *k*, *θ*, *α*);

8:Generalization;

9:**return** *T*_*i*_;

The algorithm merges records with the same caseid into super records firstly (line 1). Next, it achieves QID-bounding strategy to prevent *F*-attack (line 2-line 6). Then, the algorithm groups the records in current table with **procedure Grouping** (line 7). Last, the algorithm anonymizes the table and releases it (line 8-line 9). Our algorithm has made **three changes** to the PPMS_EAR [22]. We first redefine the privacy risk to satisfy *θ*-bounding when medication discontinuation-attack is considered (**the change 1**). The introduce and analysis of **the change 1** are as follows.

**Lemma 1.** To resist Medication discontinuation-attack, for any sensitive value *v*, the allowed largest number of *v* in a group *G* is as formula (1).


$$\eta_{v}(G)=\lfloor |GV_{1}|*\theta_{v}\rfloor$$ (1)

**Proof.** It is easy to know that *η*_*v*_(*G*)/|*GV*_1_|≤*θ*_*v*_. Meanwhile, it is clear that *η*_*v*_(*G*)/|*GV*_x_|≤*θ*_*v*_ because |*GV*_*x*_|≥|*GV*_1_| (*x*∈{2, 3, 4}). Thus, the frequency of *v* will not exceed the threshold *θ*_*v*_ in the four views of group *G*. The proof is completed.

The privacy risk is the same as that in PPMS_EAR [22] except *η*_*v*_(*G*), it is as formula (2).


$${PR}_{v}(G\cup \{t\})=\left\{ \begin{array}{l} \frac{\sigma_{v}(G\cup \{t\})}{\eta_{v}(G\cup \{t\})-\sigma_{v}(G\cup \{t\})+1},\;\sigma_{v}(G\cup \{t\})\leq \eta_{v}(G\cup \{t\})\\ \;\infty \;,\;\sigma_{v}(G\cup \{t\})>\eta_{v}(G\cup \{t\}) \end{array}\right.$$ (2)

*PR*_*v*_(*G*∪{*t*}) can evaluate the privacy risk caused by record *t*’s sensitive value *v* after *G* including *t*. The occurrence of *v* in *G* is denoted as *σ*_*v*_(*G*). In fact, a record usually has multiple sensitive values in ADE data, thus the privacy risk caused by record *t* is as formula (3).


$$PR(G\cup \{t\})=\left\{ \begin{array}{l} 1+\sum_{v\in S_{t}}{PR}_{v}(G\cup \{t\}),\;\sigma_{v}(G\cup \{t\})\leq \eta_{v}(G\cup \{t\})\\ \;\infty \;,\;\sigma_{v}(G\cup \{t\})>\eta_{v}(G\cup \{t\}) \end{array}\right.$$ (3)

*S*_*t*_ denotes the set of all the sensitive values contained by record *t*.

The difference of information loss (△*IL*(*G*, *t*)) between group *G* and group *G*∪*t* is the same as in PPMS_EAR. Thus, we can get the△*PRIL*(*G*, *t*) [22] which is as formula (4).


△PRIL(G,t)=△IL(G,t)*PR(G∪t) (4)

A record *t* has less △*PRIL*(*G*, *t*) [22] with a greater probability to be included in group *G*. This use of △*PRIL*(*G*, *t*) is shown on line 10 of the **procedure grouping** which will be introduced with **the change 2**. △*PRIL*(*G*, *t*) = ∞ represents that the inclusion of *t* will break the *θ*-bounding, thus *t* cannot be included in *G* when △*PRIL*(*G*, *t*) = ∞. Therefore, **the change 1** is actually about the redefinition of △*PRIL*(*G*, *t*) with the consideration of *MD*-attack.

Now we illustrate **the change 2** which is included by the **procedure grouping**.

**Procedure 1:** Grouping

**Input**: *T*_*i*_, *D*_*i*_, *k*, *θ*, *α*

**Output**: *T*_*i*_

/* Lines 1–24 are the steps of creating groups */

1:Randomly choose a record *t* from *D*_*i*_;

2:*D*_*i*_ = *D*_*i*_-*t*;

3:**while**(true)

4:{

5: Create a new empty group *G*;

/* Update_Group (*G*, *t*) updates *G*’s parameters which will be used by Jugde_*α*_bounding(*G*, *t*^~^, *α*). The details of the **procedure Update_Group** will be introduced later */

6: *G* = Update_Group (*G*, *t*); //*G*∪*t*_*bst*_, the parameters of *G* are updated

7: **while** (|*GV*_1_|<*k*)

8: {

/* Jugde_*α*_bounding(*G*, *t*^~^, *α*) determines whether *G*∪*t*^~^ violates *α*_bounding requirement.

Jugde_*α*_bounding(*G*, *t*^~^, *α*) = true indicates that *G*∪*t*^~^ meets the *α*_bounding requirement.

Jugde_*α*_bounding(*G*, *t*^~^, *α*) = false indicates that the *α*_bounding requirement cannot be met.

The details of the **procedure Jugde_*α*_bounding** will be introduced later. */

9: *D*_*i*_^~^contains all these records *t*^~^ in *D*_*i*_: Jugde_*α*_bounding(*G*, *t*^~^, *α*) = true;

10: Find a record *t*_*bst*_ (*t*_*bst*_∈ *D*_*i*_^~^)has the least △*PRIL*(*G*, *t*_*bst*_) in *D*_*i*_^~^;

11: **if** *t*_*bst*_ can not be found **then** // If creating groups fails

12: {

13: Add all records of *G* to *D*_*i*_;

14: Break; //The process of creating groups is completed, jumps to the line 19

15:}

16: *G* = Update_Group (*G*, *t*_*bst*_); //*G*∪*t*_*bst*_, the parameters of *G* are updated

17: *D*_*i*_ = *D*_*i*_-*t*_*bst*_;

18:} //end **while** (|*GV*_1_|<*k*)

19: **if** |*GV*_1_|> = *k* **then** // If creating groups successes

20: *T*_*i*_∪*G*;

21: **else//** If creating groups fails

22: Break;// The process of creating groups is completed, jumps to the line 25

23: Choose a record *t* which is farthest from *t*_*bst*_;

24:}//end **while**(true)

/*Lines 25–30 are the steps of processing the remaining records*/

25:**for each** *t*∈*D*_*i*_ **do**

26:{

27: Find a group *G* from *T*_*i*_ which has the least △*PRIL*(*G*, *t*);

28: *G*∪*t*;

29: *D*_*i*_ = *D*_*i*_-*t*;

30:}

In the **procedure grouping**, to resist medication discontinuation-attack, for each group *G*, the |*GV*_1_| of *G* should be no less than *k* (**the change 2**, line 7- line 18). When |*GV*_1_| = *k*, group *G* is completed; otherwise, the grouping of *G* will continue. Thus, the *k*-bounding can be guaranteed. Besides, the procedure processes the remaining records after the completion of grouping step (line 25-line 30).

**The third change** is also in **procedure grouping**. We should verify if the *α*-bounding can be satisfied. However, before *G* is completed, |*GV*_*x*_| (*x*∈{2, 3, 4}) cannot be known. Thus, we find a way to verify *α*-bounding in the group process.

**Lemma 2.** F_*ss*_(*GV*_2_) is no more than |*GV*_2_(ss)| / (|*GV*_2_- *GV*_2_(n&nmd&nss)|).

**Proof.** It is easy to know that |*GV*_2_- *GV*_2_(n&nmd&nss)|≤ |*GV*_2_|. Thus, |*GV*_2_(ss)| / (|*GV*_2_- *GV*_2_(n&nmd&nss)|)≥|*GV*_2_(ss)|/| *GV*_2_| = *F*_*ss*_(*GV*_2_).

Similarly, we can get:

F_ss_(*GV*_3_) ≤ |*GV*_3_(ss)| / (|*GV*_3_- *GV*_3_(o&nss)|).

F_ss_(*GV*_4_) ≤|*GV*_4_(ss)| / (|*GV*_4_- *GV*_4_(o&nss)- *GV*_4_(n&nmd&nss)|).

According to the above observations, it is easy to know that *GV*_2_ meets the *a*- bounding requirement when *a* ≥ |*GV*_2_(ss)| / (|*GV*_2_-*GV*_2_(n&nmd&nss)|). The similar conclusions can be got for *GV*_3_ and *GV*_4_. According to these conclusions, we can verify *α*-bounding through **procedure Jugde_*α*_bounding** and **procedure Update_Group.** These two procedures are shown below.

**Procedure 2:** Jugde_*α*_bounding

**Input**: *G*, *t*

**Output**: true or false

*G* is completed in one of these conditions:

In grouping step, the grouping of *G* is completed;

In the step of processing remaining record, a remaining record is added to *G*.

*G*.*MSAVR*_1_ represents *GV*_1_(*SS*) after group *G* is completed;

*G*.*MSAVR*_2_ represents *GV*_2_(*SS*) after group *G* is completed;

*G*.*MSAVR*_3_ represents *GV*_3_(*SS*) after group *G* is completed;

*G*.*MSAVR*_4_ represents *GV*_4_(*SS*) after group *G* is completed;

*G*.*N*_1_ represents |*GV*_1_ |after group *G* is completed;

*G*.*N*_2_ represents |*GV*_2_- *GV*_2_(n&nmd&nss) |after group *G* is completed;

*G*.*N*_3_ represents |*GV*_3_- *GV*_3_(o&nss) |after group *G* is completed;

*G*.*N*_4_ represents |*GV*_4_- *GV*_4_(o&nss)- *GV*_4_(n&nmd&nss) |after group *G* is completed;

1:When *G* is created without records, *G*.*MSAVR*_*x*_ *= 0*, *G*.*N*_*x*_ = *k*(*x*∈{1, 2, 3, 4}).

2:*G* = Update_Group (*G*, *t*);

3:**if** (*G*.*MSAVR*_1_ / *G*.*N*_1_ >*α* || *G*.*MSAVR*_2_ / *G*.*N*_2_ >*α* || *G*.*MSAVR*_3_ /*G*.*N*_3_ >*α* || *G*.*MSAVR*_4_ / *G*.*N*_4_ >*α*)

4: **return** false;

5:**else**

6: **return** true;

The **procedure Jugde_*α*_bounding** determines whether the group *G* violates *α*_bounding requirement after incorporating the record *t*. According to **procedure 1**, each group contains at least *k* n&md-records when the creation of it is completed. The set *R*_*G*_ is used to represent these *k* n&md-records of *G*. It is clear that there are *R*_*G*_ ⊆*GV*_1_, *R*_*G*_ ⊆(*GV*_2_- *GV*_2_(n&nmd&nss)), *R*_*G*_ ⊆(*GV*_3_- *GV*_3_(o&nss)) and *R*_*G*_ ⊆(*GV*_4_- *GV*_4_(o&nss)- *GV*_4_(n&nmd&nss)), when the creation of *G* is completed. Thus, we reserve the positions for the above *k* n&md-records in *GV*_*1*_, *GV*_2_-*GV*_2_(n&nmd&nss), *GV*_3_- *GV*_3_(o&nss) and *GV*_4_-*GV*_4_(o&nss)-*GV*_4_(n&nmd&nss), respectively. The initial value of *G*.*N*_*x*_ (*x*∈{1, 2, 3, 4}) is *k* (line 1). Besides, we assume that all the above *k* n&md-records are nss-records at the beginning of the creation of *G*, so the initial value of *G*.*MSAVR*_*x*_ is *0* (*x*∈{1, 2, 3, 4})(line 1). After updating *G*.*MSAVR*_*x*_ and *G*.*N*_*x*_ (line 2), the **procedure Jugde_*α*_bounding** verifies *α*_bounding: if all the four views of group *G* can meet the *α*_bounding requirement, then the *α*_bounding can be met by *G* (line 3-line 6). The main function of the **procedure Update_Group** is to update *G*.*MSAVR*_*x*_ and *G*.*N*_*x*_ after incorporating the record *t*.

**Procedure 3:** Update_Group

**Input**: *G*, *t*

**Output**: *G*

1:*G* = *G*∪*t*

2:**if** *t* is n&md-record **then**

3:{

4: **if** *t* is ss-record **then**

5: {

6: *G*.*MSAVR*_1_++; *G*.*MSAVR*_2_++; *G*.*MSAVR*_3_++; *G*.*MSAVR*_4_++;

7: **if** *t* is a remaining record **then**

8: {

/* *t*∉*R*_*G*_, *t*∈*GV*_x_(*x*∈{1, 2, 3, 4}), *t*∈(*GV*_2_-*GV*_2_(n&nmd&nss)), *t*∈(*GV*_3_- *GV*_3_(o&nss)) and *t*∈(*GV*_4_- *GV*_4_(o&nss)- *GV*_4_(n&nmd&nss)) */

9: *G*.*N*_1_++; *G*.*N*_2_++; *G*.*N*_3_++; *G*.*N*_4_++;

10:}

11:}

12}

13:**else if** *t* is n&nmd-record **then**

14:{

15: **if** *t* is ss-record **then**

16: {

/* *t*∉*R*_*G*_, *t*∈*GV*_2_, *t*∈*GV*_4_, *t*∈(*GV*_2_- *GV*_2_(n&nmd&nss)), *t*∈(*GV*_4_- *GV*_4_(o&nss)-*GV*_4_(n&nmd&nss))*/

17: *G*.*MSAVR*_2_++; *G*.*MSAVR*_4_++; *G*.*N*_2_++; *G*.*N*_4_++;

18:}

19:}

20:**else if** *t* is o&md-record **then**

21:{

22: **if** *t* is ss-record **then**

23: {

/* *t*∉*R*_*G*_, *t*∈*GV*_3_, *t*∈*GV*_4_, *t*∈(*GV*_3_- *GV*_3_(o&nss)), *t*∈(*GV*_4_- *GV*_4_(o&nss)- *GV*_4_(n&nmd&nss))*/

24: *G*.*MSAVR*_3_++; *G*.*MSAVR*_4_++; *G*.*N*_3_++; *G*.*N*_4_++;

25:}

26:}

27:**else**

28:{

29: **if** *t* is ss-record **then**

30: {

/* *t*∉*R*_*G*_, *t*∈*GV*_4_, *t*∈(*GV*_4_- *GV*_4_(o&nss)- *GV*_4_(n&nmd&nss))*/

31: *G*.*MSAVR*_4_++; *G*.*N*_4_++;

32:}

33:}

34:**return** *G*;

For various types of records, the **procedure Update_Group** updates *G*.*MSAVR*_*x*_ and *G*.*N*_*x*_ (*x*∈{1,2,3,4}). Now we take the lines 2–12 as an example. These lines consider *G*.*MSAVR*_*x*_ and *G*.*N*_*x*_ when *t* is a n&md-record. It is clear that there is *t*∈*GV*_*x*_(*x*∈{1,2,3,4}) when *t* is a n&md-record. Thus, *G*.*MSAVR*_*x*_ is updated if *t* is an ss-record (line 6). On the other hand, the positions of *t* have been already reserved in *GV*_*1*_, *GV*_2_-*GV*_2_(n&nmd&nss), *GV*_3_-*GV*_3_(o&nss) and *GV*_4_-*GV*_4_(o&nss)-*GV*_4_(n&nmd&nss), when *t* is not a remaining record. Therefore, *G*.*N*_*x*_ (*x*∈{1,2,3,4}) remains unchanged when *t* is not a remaining record. However, there are *t*∉*R*_*G*_, *t*∈*GV*_1_, *t*∈(*GV*_2_-*GV*_2_(n&nmd&nss)), *t*∈(*GV*_3_-*GV*_3_(o&nss)) and *t*∈(*GV*_4_-*GV*_4_(o&nss)-*GV*_4_(n&nmd&nss)) when *t* is remaining record and ss-record. Thus, *G*.*N*_x_ (*x*∈{1,2,3,4}) is updated (line 9). Similarly, the other parts of **procedure Update_Group** update *G*.*MSAVR*_*x*_ and *G*.*N*_*x*_ according to the type of *t*.

Our algorithm quits predetermining the maximum number of ss-records, so it is more flexible. The experimental results also show that our heuristic algorithm can maintain the usability of released tables when has more stringent privacy requirement.

## 5. Experimental results and analysis

In this section, we compare our method (**PPMS(*k*, *θ*, *ɑ*)-bounding** achieved by **HA**) with **PPMS_Ear** [22]. We implement both the methods with Microsoft Visual C++ 2015. All the experiments are conducted on a PC with Intel Core 2.60 GHz CPU and 8 GB main memory, running the Microsoft Windows 10 operating system.

We analyze the methods from security and information loss. The 14 recent datasets are chosen from FEARS of FDA:2014Q3-2017Q4. The quasi-identifiers (QIAs) and sensitive attributes(SAs) are the same as in [22], QIAs = {Weight, Age, Gender}, SAs = {INDI_PT, PT}. To define ss-record, we calculate the average count *AVG*_INDI_PT_/*AVG*_PT_ of INDI_PT/PT values and the corresponding standard deviation *SD*_INDI_PT_/ *SD*_PT_. We define record *t* is a **ss-record**, if *t* satisfies one of these conditions:

1. The count of INDI_PT values of *t* is no less than *AVG*_INDI_PT_+*SD*_INDI_PT_;
2. The count of PT values of *t* is no less than *AVG*_PT_+*SD*_PT_.

### 5.1 Security

Dangerous Identity Ratio(DIR) [21–22] and Dangerous Sensitivity Ratio (DSR) [21–22] are used to evaluate the security of publishing methods. We call a group as a dangerous identity group (DIG) if the number of records in the group is less than threshold *k*. If a group contains at least one sensitive value *v*_*i*_ whose frequency is higher than its threshold *θ*_*i*_, we call it as a dangerous sensitivity group (DSG). DIR/DSR represents the ratio of DIG/DSG in all anonymous groups.

For measuring the ability to resist substantial symptoms-attack, we define the substantial symptoms group ratio (SSGR). If the frequency of ss-record in a group is higher than threshold *ɑ*, we call the group as substantial symptoms group(SSG). Similar to DIR and DSR, SSGR represents the ratio of SSG in all anonymous groups.

**Fig 1:**
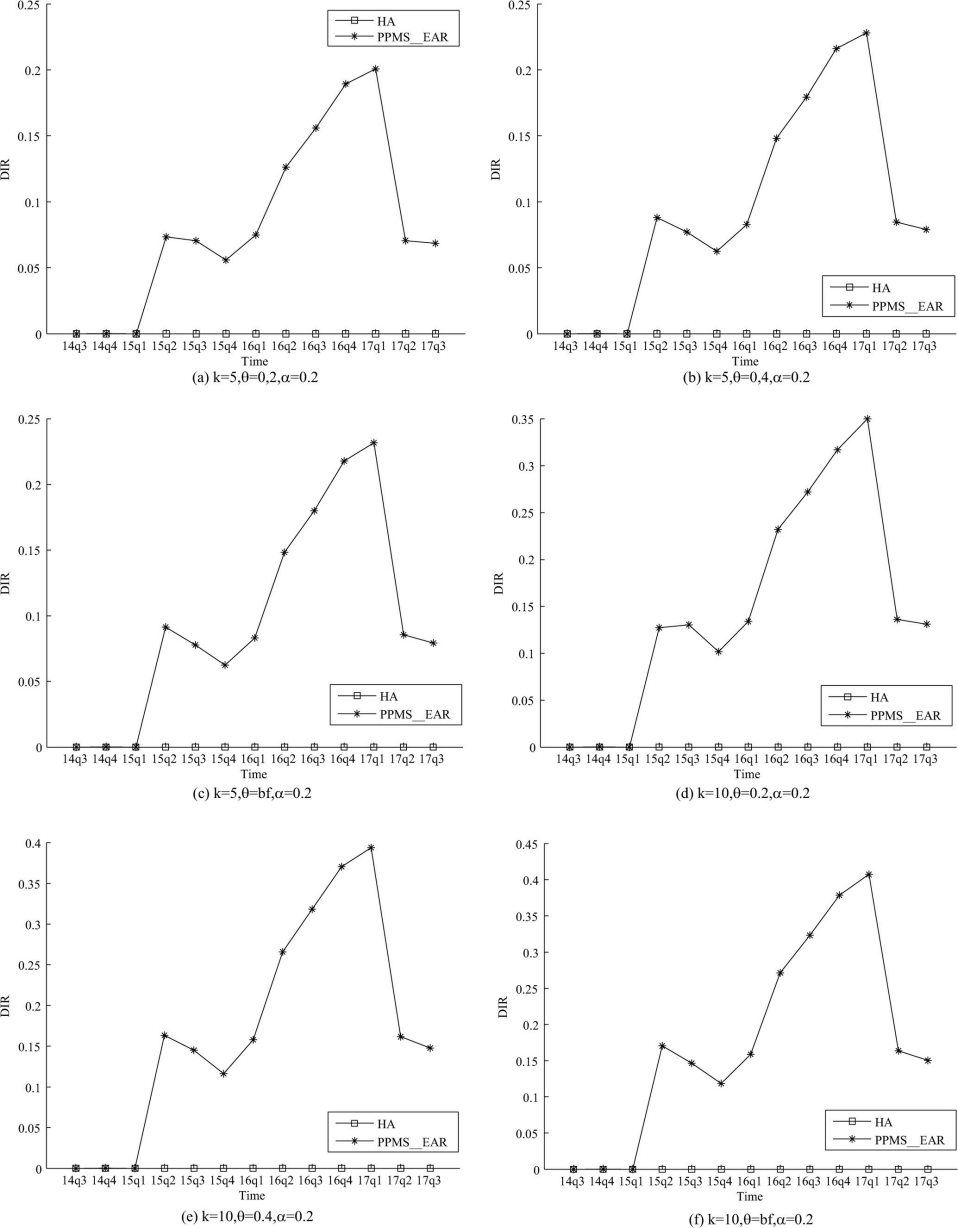
Evaluation on DIR of two methods.

**Fig 2:**
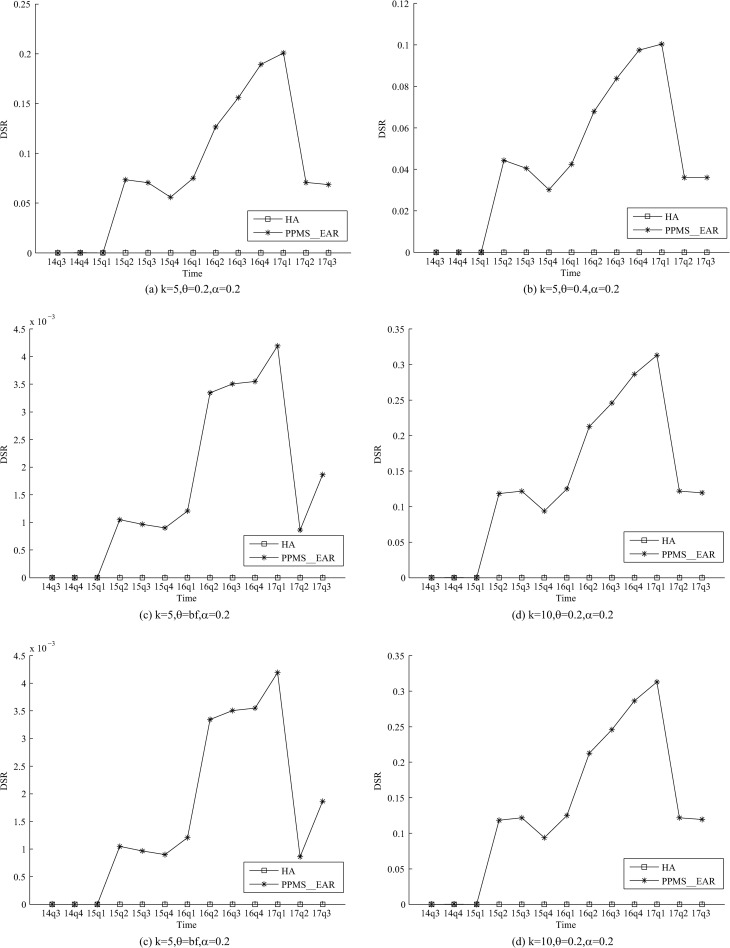
Evaluation on DSR of two methods.

As shown in Figs 1 and 2, the DIR and DSR of PPMS_Ear are both greater than 0, because PPMS_Ear has not taken the medication discontinuation-attack into consideration. The DIR and DSR are even greater than 10% in some released tables of PPMS_Ear. An adversary can compromise the privacy requirements in the released tables of PPMS_Ear which is vulnerable to the medication discontinuation-attack. lMeanwhile, PPMS(*k*, *θ*, *ɑ*)-bounding has considered that an adversary may disclose privacy through information about medication discontinuation. Therefore, PPMS(*k*, *θ*, *ɑ*)-bounding can avoid Medication discontinuation-attack, DIR and DSR are both 0.

**Fig 3:**
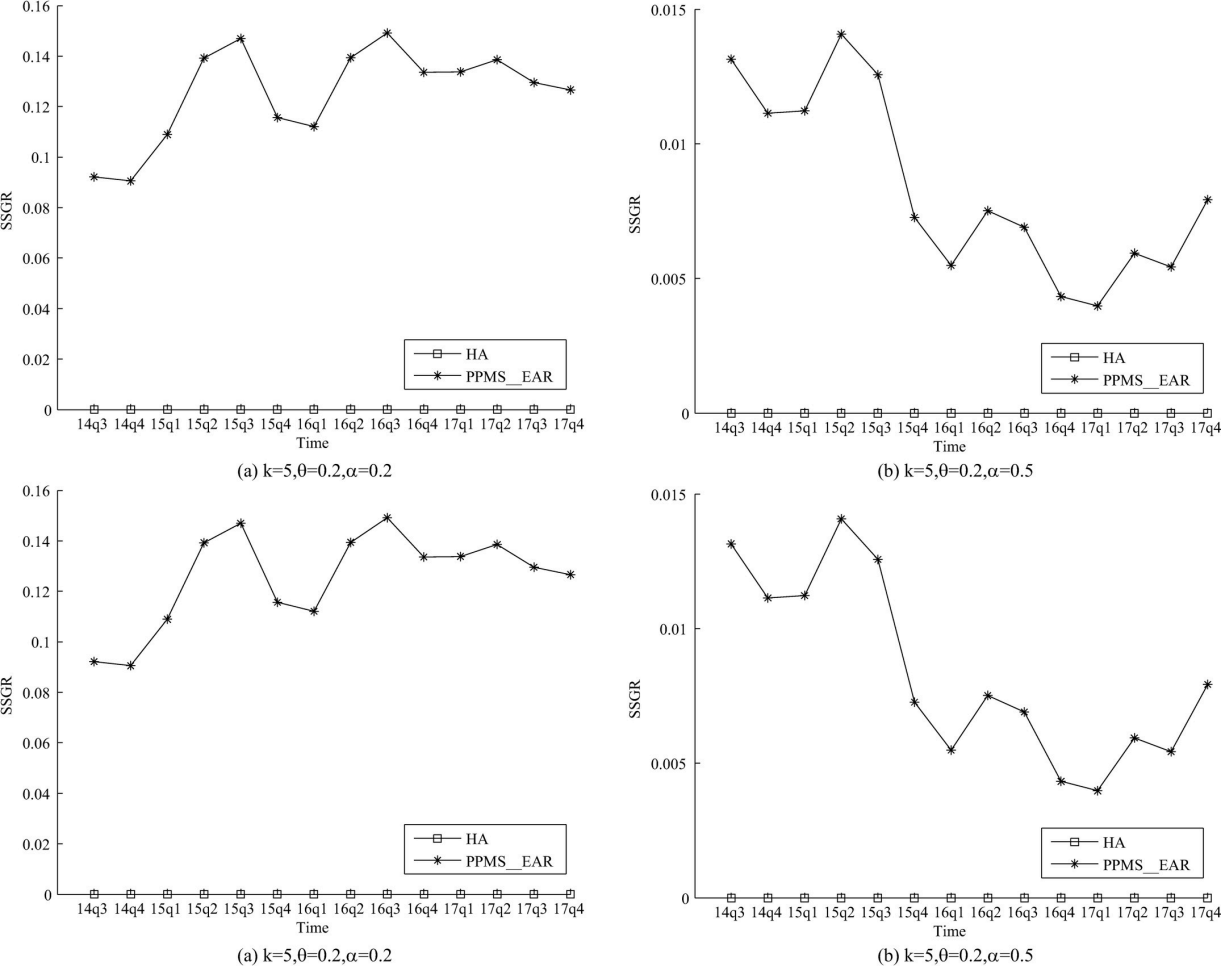
Evaluation on SSGR of two methods.

The SSGR of the two methods is shown in Fig 3. We can see that the SSGR of PPMS(*k*, *θ*, *ɑ*)-bounding is 0 because our method can thwart *SS*-attack with limiting the frequencies of ss-records. However, PPMS_Ear cannot resist *SS*-attack, hence its SSGR in some released tables is greater than 10%. The settings of *θ*(*θ* = 0.4, *θ* = bf) are omitted, because they generate similar results.

### 5.2 Information loss

**Fig 4:**
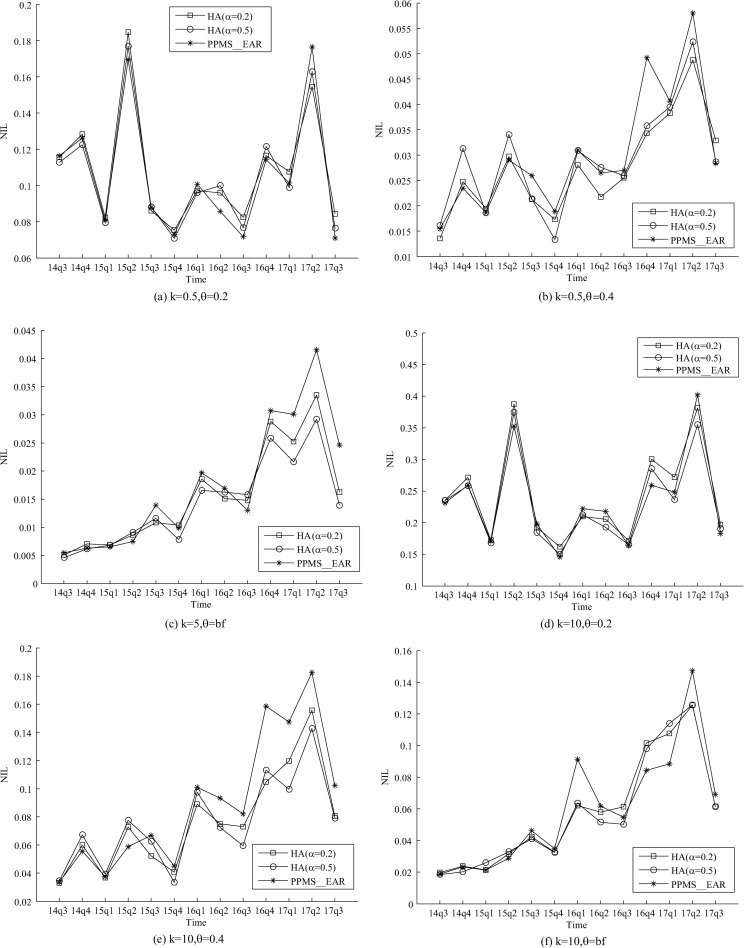
Evaluation on NIL of two methods.

Normalized Information Loss(NIL) [21–22] is used to evaluate the information usability. As shown in Fig 4, we can see that the NIL of PPMS(*k*, *θ*, *ɑ*)-bounding is very close to PPMS_Ear. Our analysis result is that **procedure Jugde_*α*_bounding** achieves *ɑ*-bounding through a heuristic method, and it is easier for records to be incorporated by groups in this method. The **HA** estimates the frequencies of ss-records to guarantee *α*-bounding when grouping. Compare with predetermining the maximum number of ss-records in groups, this heuristic method can “accommodate” more ss-records in each anonymous group while the privacy requirement is not compromised. Therefore, our method can have similar information loss with PPMS_Ear even if the privacy requirements of ours are more stringent.

## 6. Discussion

The tradeoff between privacy and utility is the focus in data publishing. The traditional periodical data publishing mechanisms [9–14] are not suitable for the SRS data due to its some special features. The existing SRS data publishing methods [22–25] had considered these types of attacks in the scenario of SRS data publishing: *B*-attack, *F*-attack and *L*-attack, trying to guarantee the information usability of the released tables. However, the SRS data has some special features, such as identity preservation and multivalued sensitive attributes, which make it more vulnerable to security threats than the other types of data. We discover and define two new attacks in this paper: *MD*- attack and *SS*-attack, and find that the existing SRS data publishing methods cannot resist them. In order to solve these problems, we present a new SRS data publishing model and the corresponding heuristic algorithm in this paper. We consider these attacks in the evaluations of DIR and DSR: *MD*-Attack, *B*-attack, *F*-attack and *L*-attack, the related experimental results (Figs 1 and 2) show that all these attacks can be resisted by the proposed method. The evaluation of SSGR is used to analyze the *SS*-attack, and the corresponding results (Fig 3) also demonstrate that the proposed method can thwart this type of attack. Thus, the results of security evaluations exhibit that the proposed method can defend against the various known attacks. For the information usability, the related experimental results (Fig 4) demonstrate that the information loss degree of the proposed method is similar to the existing one. The results of information usability evaluation suggest that the proposed heuristic algorithm is an effective way to decrease the information loss when the anonymous standard becomes more stringent.

According to the above experimental results of security and usability, we can know that the proposed method can provide better protection than the exiting SRS publishing methods and the guaranteed information usability can be provided by our method. Compared with the existing SRS publishing methods, our method can achieve a better balance between privacy security and information usability.

## 7. Conclusion

In this paper, we consider medication discontinuation and substantial symptoms in periodical ADEs data publishing, and propose a new periodical ADEs data publishing method, which can resist medication discontinuation-attack and substantial symptoms attack. The experimental results also show that our method can protect against various known attacks, and can enhance the security based on PPMS_Ear. Besides, comparing with PPMS_Ear, our method does not have an obvious increase in information loss.

Several directions for future work are also initiated by this work. First, it would be interesting to study personalized anonymity [26] in SRSs data publishing. This technique enables individuals to specify privacy levels to their own sensitive information, in order to yield a better tradeoff between privacy security and information utility. Second, it would be exciting to extend the proposed method to be applicable for big data publication [27]. Research towards this direction may discover effective parallel algorithms with guaranteed privacy security and information usability.
